# Is mushroom polysaccharide extract a better fat replacer than dried mushroom powder for food applications?

**DOI:** 10.3389/fnut.2023.1111955

**Published:** 2023-02-03

**Authors:** Cheryl Jie Yi See Toh, Xinyan Bi, Hui Wen Lee, Michelle Ting Yun Yeo, Christiani Jeyakumar Henry

**Affiliations:** ^1^Clinical Nutrition Research Centre (CNRC), Singapore Institute of Food and Biotechnology Innovation (SIFBI), Agency for Science, Technology and Research (A*STAR), Singapore, Singapore; ^2^Department of Biochemistry, Yong Loo Lin School of Medicine, National University of Singapore, Singapore, Singapore

**Keywords:** mushrooms, polysaccharide, fat replacement, commercial application, GC-MS, dietary fiber, functional food (FF)

## Abstract

**Introduction:**

β-glucans found in the cell walls of mushrooms can be a beneficial food additive in replacing fat in commercial food products.

**Methods:**

Four commonly consumed mushroom species in Singapore, i.e., *Pleurotus ostreatus* spp., *Lentinus edodes*, *Agaricus bisporus*, and *Flammulina velutipes* were profiled for the β-glucan content in the lyophilized form and ultrasonicated assisted extracted form. Both forms were added into chicken patties, which were characterized for the moisture, cooking loss, texture, color, and chemically analyzed for the protein, crude fat, and fatty acid profiles with gas chromatography mass spectrometry (GC-MS).

**Results and discussion:**

*Pleurotus Ostreatus* spp. had the highest β-glucan of 29.8 ± 0.7 g/100 g in the pure powder form and 15.9 ± 0.3 g/100 g from the extract. Crude fat in 100% fat substituted patties was lowest in *Flammulina velutipes* extract enriched patties and least in *A. bisporus* pure powder patties. Additionally, fat replacement with *A. bisporus* extract and powder forms resulted in the highest polyunsaturated fatty acid profile of 49.6 ± 1.9 mg/100 g patty and 79.9 ± 4.5 mg/100 g patty, respectively. Chicken patties with added mushroom extract were notable in retaining moisture, cooking yield and its structure. Fat substitution with mushroom powder was also conducted, satisfactory results indicated a possibility as a better fat replacer that is easily processed and an efficient alternative to β-glucan extract. With increasing demand for low fat foods with acceptable organoleptic properties, our study demonstrates that the inclusion of dry mushroom powder has the ability to mimic the “fattiness” of chicken patties.

## 1. Introduction

Mushrooms are fungi that are highly prized not only for its flavor and texture added in foods but also known as a superfood that possess nutritional and medicinal benefits. Mushrooms are low in calories, fat, and cholesterol, but high in dietary fiber (1, 2). In addition, mushrooms have been known to contain high levels of monounsaturated (MUFAs), polyunsaturated (PUFAs), and moderate amounts of saturated fatty acids (SFAs). Mushrooms are a source of essential fatty acids, such as linoleic and linolenic fatty acids that are not produced by the human body (3). Fatty acids are important in human health for immune function, and can reduce metabolic disease biomarkers, however, the type of predominant fatty acids in the diet should be PUFAs for these benefits (4). Consumers’ demand in low fat meat products is on the rise driving the food industry to find ways to reduce SFAs and trans fats whilst increasing MUFAs and PUFAs in commercial meat products through the addition of dietary fiber and whey additives (5).

β-glucans are a form of dietary fiber, well known to have anti-diabetic, anti-cancerous, cholesterol- and glycogen-lowering properties, aiding in the prevention of non-communicable diseases (6–8). Although β-glucans are found in many different sources including oats, yeast, barley, and bacteria, mushroom β-glucan differs from wheat derived β-glucan for its unique β-(1, 3) linkages attached to branches with β-(1, 6) linkages and are mainly found in abundance within the mushroom cell walls (9). Previous studies have shown that mushroom polysaccharide extract contained valuable antioxidants and bioactives other than triterpenes that stimulated glucose uptake to improve insulin sensitivity in diabetic mice (10).

Excessive consumption of a high fat, and high caloric diet has been shown to increase the risk of obesity and type 2 diabetes (11). Researchers and food companies worldwide have been engaged in trying to reduce the cholesterol and lipid contents in a high fat diet. Previously, mushroom powders incorporated in meat products have been shown to improve dietary fiber content, reduce fat content while maintaining textural properties and sensory attributes (12, 13). Nonetheless, fats play a key role in diets to ensure optimal rheological and textural properties and to confer pleasant sensorial characteristics (14, 15). Consequently, β-glucan polysaccharides have been gaining popularity as fat-replacement ingredients to create low-fat products that mimic commercial food products whilst maintaining its textural, structure, sensory properties and imparting additional nutritional qualities (13, 16). Sensory attributes such as the color of meat products provide a visual aid in determining the safety, acceptability, and taste perception of the food (17).

Although β-glucans show useful properties which make them promising ingredients in health-promoting functional foods, to the best of our knowledge, mushroom β-glucan extracts have not been investigated in a food matrix to determine its feasibility as a beneficial food additive. Moreover, the amount and yield of β-glucan vary between different sources of mushrooms as well as its extraction techniques (18). Previously, several extraction methods including hot water extraction, ultrasonic assisted extraction (UAE), microwave extraction and supercritical fluid CO_2_ extraction have been successfully employed to extract β-glucan from mushroom (19). Among them, UAE is efficient in breaking down β-glucan from mushroom cell walls, uses less solvent while maintaining high product yield and a stronger polysaccharide matrix (20). Furthermore, β-glucan extraction eliminates fat bound to mushrooms and has been found to limit fat absorption in mice (21). Therefore, mushroom β-glucans can be a source of dietary fiber added into meat patties as a fat replacer to reduce its fat content while promoting a favorable fatty acid profile.

In this study, the availability of crude β-glucan from five common mushrooms, i.e., oyster (*Pleurotus ostreatus* spp.), enoki (*Flammulina velutipes*), shiitake (*Letinus edodes*), white button (*Agaricus bisporus*), and mini portobello (*Agaricus bisporus*) mushroom, widely available and consumed in Singapore was determined using UAE and ethanol precipitation. Both the extracted β-glucan and dried mushroom powder were added into chicken meat patties as a potential fat replacer, respectively, and tested for the physicochemical characteristics of moisture, cooking changes, texture, protein, and crude fat content. Further determination of its fatty acid profile was attained by gas chromatography mass spectrometry (GC-MS).

## 2. Materials and methods

### 2.1. Materials and chemicals

Five types of mushrooms, oyster (Pasar, Malaysia), enoki (Pasar, Malaysia), shiitake (Chef, China), white button (Pasar, Holland), and mini portobello (Pasar, Malaysia), and chicken breast (Pasar, Malaysia) were purchased from a local supermarket (NTUC Fairprice, Singapore). Food grade ethanol (Echo Chemical Co., LTD, China) was used for extraction. Methyl undecanoate (C-11) standard, boron trifluoride (BF3), sulfuric acid, hydrochloric acid (HCl), sodium methoxide, methyl tert-butyl ester, sodium citrate dibasic sesquihydrate, GC-grade methanol and hexane were purchased from Sigma-Aldrich (St. Louis, MO, USA) along with petroleum ether (PE) (VWR, Singapore). Digestion tablets (Kjeltabs S-3.5, Foss™) for protein determination was purchased from Nexus Analytics (Singapore).

### 2.2. Mushroom powder preparation

Mushrooms were wiped cleaned with kitchen paper, then sliced into smaller pieces and lyophilized with VirTis Ultra 35 L pilot lyophiliser (SP Scientific Products; Stone Ridge, NY, USA). Lyophilized mushrooms were blended in a warring grinder (Waring^®^ WSG60, USA) and sifted with a kitchen sift before storage in the dark at −80°C.

### 2.3. UAE extraction

Extraction steps were modified based on previous protocols (22). Briefly, 10 g of powdered mushroom was added into 340 ml water and placed in an ultrasonic bath (FB15055, Fisherbrand^®^, Fisher Scientific Pte Ltd, Singapore) at 50°C for 30 min (≤550 W). The mixture was centrifuged at 7,981 × *g* at 4°C for 15 min. The supernatant was collected and reduced to 70 ml in a rotary evaporator (Rotavapor^®^ R100, BÜCHI Labortechnik, Switzerland) with a heating water bath and condenser temperatures of 60 and 10°C, respectively. The supernatant was added to four-fold volumes of 100% ethanol and precipitated overnight at 4°C. After centrifuging the suspension at 7,981 × *g* at 4°C for 15 min, the supernatant was removed to collect the precipitated extract. The extract was washed twice with 100% ethanol and vacuum dried with the Vacucell 55 EVO vacuum oven (MMM Group, Germany) at 30°C and 178 mbar overnight. Dried extract was ground with a mortar and pestle, then stored at −80°C until used.

### 2.4. Preparation of chicken patties

Formulations of β-glucan patties were adapted from Wan-Mohtar et al. (23). Chicken breast was prepared by removing excess visible fat and minced in a mincer (Grindomix GM 200, Retsch, Germany) at 5,000 rpm for 10 s. Next, the remaining ingredients were manually mixed for 3 min (Table 1). Mixture from each formulation was formed into 10 g patties with a 3 × 3 × 1 cm mold and stored at −20°C freezer overnight. Patties were thawed before cooking in a non-stick pan on an induction hob (PIE645F17E Ceramic Induction Hob, Borsch, Germany) for 3 min on each side. Cooking weight and dimensions were recorded before and after cooking from thawed patties. Similar procedures were followed for the mushroom powder patties (Table 2) with modifications based on β-glucan patties and adaptations from Wan Rosli et al. (24). Mixtures from each formulation were formed into 50 g patties with an 8 × 8 × 1 cm mold and placed at −20°C overnight before thawing and cooking. Cooking loss was calculated as percent weight loss in cooked samples from raw samples (Eq. 1), while shrinkage was calculated as percent dimension loss in cooked samples from original uncooked frozen samples (Eq. 2).


(1) $$\mathrm{Cooking}\mathrm{Loss}\left(\%\right)=\frac{\mathrm{Raw}\,\mathrm{Thawed}\,\mathrm{Weight}-\mathrm{Cooked}\,\mathrm{Weight}}{\mathrm{Raw}\,\mathrm{Thawed}\,\mathrm{Weight}}\times 100$$

(2) $$\text{Shrinkage}\left(\%\right)=\frac{\mathrm{Original}\,\mathrm{Dimension}-\mathrm{Cooked}\,\mathrm{Dimension}}{\mathrm{Original}\,\mathrm{Dimension}}\times 100$$

**TABLE 1:** Formulation (g/100 g patties) of three different chicken patties without fat substitution (CTRL), as well as at 50% (T1) and 100% (T2) fat substitution with β-glucan polysaccharide extract.

Ingredients	CTRL	T1	T2
Chicken breast	92	92	92
Soybean oil	2.5	1.25	0
β-glucan	0	1.25	2.5
Salt	0.25	0.25	0.25
Seasoning (pepper)	0.25	0.25	0.25
Potato starch	3	3	3
Water	2	2	2.5

**TABLE 2:** Formulation (g/100 g patties) of three different chicken patties without fat substitution (CTRL_M), as well as at 50% (T1_M) and 100% (T2_M) fat substitution with freeze-dried mushroom powders.

Ingredients	CTRL_M	T1_M	T2_M
Chicken breast	80	80	80
Soybean oil	10	5	0
Mushroom powder	0	5	10
Salt	0.4	0.4	0.4
Seasoning (pepper)	0.6	0.6	0.6
Potato starch	4	4	4
Water	5	5	5

### 2.5. Texture, color, and moisture analysis

Cooked patties were cut into small pieces with the dimension of 1.5 × 1.5 × 1 cm and tested for a two-bite texture analysis (TA.XT plus, Stable Micro Systems, UK) with a P/75 probe at 50% strain at 1 mm/s for the first compression before retracting to baseline set by 5 s, followed by the subsequent second bite compression at the same conditions (25). Hardness and chewiness were measured and averaged from duplicate samples. The formula for hardness and chewiness as well as an example graph of the two bite test can be found in the Supplementary materials page. Cooked samples were distributed into a glass petri-dish and measured with a colorimeter (Spectrophotometer CM-5, Konica Minolta, Japan). Color analysis was performed for mushroom powder patties and averaged from triplicate readings taken from three samples. The measurements were made in specular component excluded (SCE) mode, D65 illuminant, 10° observer angle and a measurement area of 30 mm. Results were expressed in L* for darkness to lightness, a* for redness, and b* for yellowness (26). Total color difference was calculated by Delta E (ΔE) value from the L*, a*, and b* of the control (CTRL_M) as compared to the other mushroom powder patties (Eq. 3). The patty samples were then dried in an oven at 105°C for 24 h for moisture content analysis. After drying, samples were ground into powder with a spice grinder (EGK 200, Rommelsbacher, Germany) and stored in a dark desiccator at room temperature.


(3) △E =(L*2-L*1)2+(a*2-a*1)2+(b*2-b*1)2

### 2.6. Protein analysis

Total nitrogen in patties and freeze dried mushroom powder were determined with the Kjeldahl method adapted from Chiang et al. (25) using a Tecator™ Digestor 8, Tecator™ Scrubber, and Kjeltec™ 8200 Auto Distilling Unit (FOSS Analytical, Hoganas, Sweden). Dried patty samples (0.2 g) and mushroom powder (1 g) were analyzed in duplicate. Two Kjeltabs selenium tablets and 15 ml of concentrated sulfuric acid were added into each sample for digestion at 401°C for 1–2 h till the digestate became clear. After cooling, the digestate underwent distillation with 70 ml of water and 50 ml of sodium hydroxide (40% w/v) and 25 ml of boric acid (4% w/v) was used as the trapping solution for the liberated ammonia gas. Samples were then titrated to equivalence with 0.1 M HCl, and the amount of HCl used was recorded. Protein content was then calculated by a nitrogen conversion factor of 6.25 (13) for meat patty samples and 4.38 (2) for pure mushroom powder samples.

### 2.7. Crude fat analysis

Crude fat extraction protocol was adapted from Yeo et al. (27) using Soxtec extraction method (Soxtec™ 2055, Foss™, Denmark). Dried sample (2.5–3 g) was weighed in duplicates into individual thimbles and covered with de-fatted cotton, before oven dried at 103°C for 2 h and cooled for 30 min in a desiccator. Corresponding thimbles and sample cups were set into the apparatus with 80 ml of petroleum ether added to each sample cup. Crude fat was extracted at 135°C at a boiling time of 20 min, rinsing time of 40 min for recovery and drying time of 10 min, respectively. After extraction, sample cups were dried in an oven at 103°C for 30 min to evaporate the remaining solvent before weighing of cups and quantification of crude fat. Crude fat was stored at −80°C before derivatisation for fatty acid identification.

### 2.8. Fatty acid methyl ester (FAME) analysis

Crude fat extracted from β-glucan patties were derivatized with procedures adapted from Yeo et al. (28). Crude fat (25 ± 1 mg) was spiked with 15 μl of C-11 internal standard before an addition of 1 ml of 2 N NaOH in methanol. The mixture was vortexed till no residue remained and placed in a water bath at 80°C for 1 h. After cooling, 1 ml of 25% (v/v) boron trifluoride in methanol was added and mixed well before incubation at 80°C water bath for another 1 h. After cooling, 2.5 ml of water and hexane were added, respectively, and the mixture was vortexed before allowing to stand for the separation of the two phases. The top organic layer was transferred into a GC vial for FAME analysis.

Mushroom powder patties were derivatized based on Golay et al. and Agilent technical note (29, 30). Dried powder patties (25 ± 1 mg equivalent of fat based on determined crude fat) were mixed with 1 ml of MilliQ water, vortexed and left to stand at room temperature for 15 min. Subsequently, 2.5 ml of C-11 internal standard solution (0.6 mg/ml) and 2.5 ml of 5% (v/v) methanolic sodium methoxide was added, vortexed for 10 s and left to stand for 180 s. Next, 1 ml of hexane was added and left to stand for an additional 210 s. Finally, 5 ml of disodium hydrogen citrate and sodium chloride mixture was added and vortexed well. The mixture was then transferred into a 50 ml centrifuge tube for centrifugation at 375 × g for 5 min and the top layer was transferred into a GC vial for (FAME) analysis.

Fatty acid analysis was determined by GC-MS with a 7890 B GC system coupled to a 5977 B MS detector (Agilent Technologies, Santa Clara, CA, USA). FAME separation procedures were adapted from Shun and Yun (31) using an Agilent HP-88 column (100 m × 0.25 mm, 0.20 μm) with the split injector maintained at 260°C and a spilt ratio of 50:1. The oven temperature program was as follows: isothermal at 140°C for 5 min and increased to 240°C at a rate of 4°C/min. Helium was used as the carrier gas under a constant flow mode of 1 ml/min. Results were analyzed by comparison with the reference standard GLC-36 (Nu-Chek Prep, USA) and the NIST library (NIST 14) using ChemStation software (MSD ChemStation F.01.03.2357).

### 2.9. Beta-glucan analysis

Mushroom β-glucan was determined with the Megazyme K-YBGL yeast and mushroom β-glucan assay kit (Megazyme Ltd, Wicklow, Ireland). Freeze dried mushrooms, dried crude polysaccharide extracts, and dried β-glucan polysaccharide meat patty samples were analyzed in duplicates with procedures adapted from Megazyme assay protocol (32).

For total glucan content estimation, 45 mg of dried sample was mixed with 1 ml of 12 M sulphuric acid, vortexed, and incubated in an ice bath for 2 h with periodic mixing. Next, 5 ml of water was added and mixed for 10 s before incubation at 100°C for 2 h. After cooling, tube contents were transferred quantitatively into a 50 ml volumetric flask, with 3 ml of 8 M NaOH added and adjusted to volume with 200 mM sodium acetate. Contents were thoroughly mixed, and a 1.5 ml aliquot was collected in a 2 ml microfuge tube for centrifugation at 15,700 × *g* for 5 min. Next, 50 μl of supernatant was mixed with 50 μl of exo-1,3-β-glucanase and β-glucosidase in 200 mM sodium acetate buffer then incubated at 40°C for 1 h. Next 1.5 ml of GOPOD reagent was added and incubated further at 40°C for 1 h. Finally, absorbance was measured in a dark room with a spectrophotometer (Shimadzu UV-2600 single monochromator UV-Vis, Shimadzu, Japan) at 510 nm against a reagent blank consisting of 100 μl 200 mM sodium acetate buffer and 1.5 ml GOPOD reagent.

For α-glucan content estimation, all samples were tested according to <10% α-glucan content assay procedure. Dried sample (50 mg) was mixed with 1 ml of 1.7 M NaOH over magnetic stirring in an ice bath for 20 min. Then, 4 ml of 1.2 M sodium acetate buffer was added, followed by 10 μl of amyloglucosidase. The mixture was then incubated at 40°C water bath for 30 min with frequent mixing. Next, 2 ml of mixture was transferred into a microfuge tube and centrifuged at 15,700 × *g* for 5 min. The supernatant (100 μl) was mixed with 100 μl of 200 mM sodium acetate buffer and 3 ml of GOPOD reagent before incubation at 40°C for 20 min. Finally, absorbance was measured at 510 nm against the reagent blank. Total and α-glucan results were inserted into Mega-Calc™ spreadsheet (Megazyme Ltd, Ireland) and β-glucan was determined by subtraction of α-glucan from total glucan content.

### 2.10. Statistical analysis

Statistical analysis was performed using the SPSS version 23 (IBM, Armonk, NY, USA). All data were expressed as means ± SD (standard deviation). One way ANOVA was used for between-group comparisons (chicken patties with 50% fat substitution vs. chicken patties with 100% fat substitution). Dunnett *t*-tests were used for between-group comparisons by treating chicken patties without fat substitution as the control group and compare all other groups against it. Two-sided *p* < 0.05 was considered statistically significant in all cases.

## 3. Results

### 3.1. Chemical composition of mushrooms

In this study, we found that the five types of mushrooms from four genera, i.e., oyster mushroom (*P. ostreatus* spp.), enoki (*F. velutipes*), shiitake (*L. edodes*), white button and mini portobello (*A. bisporus*), have a high moisture content ranged from 86.4 to 93.6%, with the highest from *A. bisporus* group (Table 3). Protein content of mushrooms were moderate, the lowest was enoki mushroom at 13.1% and highest was from mini portobello at 26.5%. β-glucan approximation from freeze-dried mushroom powder found that oyster mushroom had the highest content of β-glucan [29.8 g/100 g dried matter (DM)] followed by shiitake mushroom (22.4 g/100 g DM). White button and mini portobello had relatively low contents of β-glucan (9.8 and 9.2 g/100 g DM, respectively).

**TABLE 3:** Moisture (%), crude polysaccharide yield (%), and β-glucan (g/100 g mushroom powder) content of five different mushrooms from freeze-dried powder.

Mushrooms	Moisture (%)	Crude polysaccharide (g/10 g freeze-dried mushroom)	Polysaccharide yield (%)		β-glucan (g/100 g DM)	Protein (%)
			Dry basis	Fresh basis		
Oyster (O)	90.0 ± 0.1	1.6 ± 0.0	16.0 ± 0.1	1.6 ± 0.0	29.8 ± 0.7	17.5 ± 0.1
Enoki (E)	88.2 ± 0.6	1.3 ± 0.4	12.1 ± 3.6	1.4 ± 0.4	17.1 ± 1.7	13.1 ± 0.1
White button (WB)	92.4 ± 0.6	1.9 ± 0.9	19.1 ± 9.4	1.4 ± 0.7	9.8 ± 0.5	23.0 ± 0.1
Mini portobello (MP)	93.6 ± 0.1	2.1 ± 0.1	20.8 ± 1.5	1.2 ± 0.1	9.2 ± 0.5	26.5 ± 0.0
Shiitake (S)	86.4 ± 0.3	1.5 ± 0.1	14.5 ± 1.7	2.0 ± 0.2	22.4 ± 0.2	18.4 ± 0.2

DM, dried matter.

**FIGURE 1:**
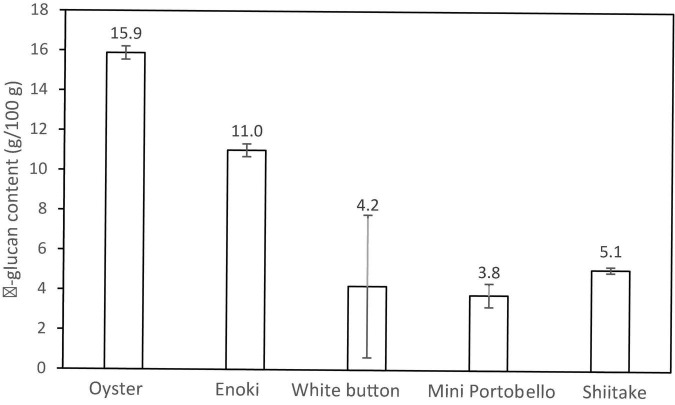
β-glucan content (g/100 g) in crude mushroom polysaccharide extract.

UAE extraction of β-glucan containing polysaccharide from fresh mushroom was low amongst the four groups, with the highest yield from mini portobello at 2.1 g/10 g and lowest yield from shiitake mushroom at 1.5 g/10 g (Table 3). β-glucan approximation from UAE polysaccharide extract was highest in oyster mushroom (15.9 g/100 g DM) followed by enoki mushroom (11.0 g/100 g DM), shiitake (5.1 g/100 g DM), white button mushroom (4.2 g/100 g DM) and mini portobello mushroom (3.8 g/100 g DM) with the least β-glucan within its polysaccharide extract (Figure 1).

### 3.2. Chemical composition of mushroom extract and powder patties

Chemical composition of chicken patties with and without fat substitution with crude mushroom polysaccharide extracts are shown in Table 4. In general, chicken patties with fat substitution had a slightly higher moisture retention (66.8 to 69.6%) than the control without fat substitution (CTRL). Upon cooking, polysaccharide substituted patties had significantly less cooking losses (9.5 to 16.7%) as compared to CTRL (*p* < 0.05 or *p* < 0.005). Moreover, the chicken patties with fat substitution had significantly lower crude fat content (1.5 to 6.9%) than CTRL (*p* < 0.005). However, protein content of the fat substituted extract patties ranged from 75.7 to 81.5%, slightly higher than CTRL at 75.2%. In addition, 100% fat substitution with crude polysaccharide extracts (T2) had a higher moisture retention, higher protein content, lower cooking loss, and lower fat (*p* < 0.005) as compared to patties with 50% fat substitution (T1).

**TABLE 4:** Chemical composition (g/100 g DM), cooking loss (%), and β-glucan content (g/100 g FW) of different chicken patties without fat substitution (CTRL), as well as at 50% (T1) and 100% (T2) fat substitution with β-glucan polysaccharide extracted from different mushrooms.

	Moisture (%)	Cooking loss (%)	Protein (g/100 g DM)	Crude fat (g/100 g DM)
CTRL	66.4 ± 0.1	21.7 ± 1.5	75.2 ± 0.3	9.3 ± 0.0
T1_O	66.8 ± 1.0	16.7 ± 0.2^a^	75.7 ± 0.3	6.9 ± 0.2^b^
T2_O	66.7 ± 0.1	13.4 ± 0.8^b,c^	78.1 ± 0.7	2.7 ± 0.0^b,d^
T1_E	68.1 ± 0.4	13.0 ± 2.2^b^	79.4 ± 0.4	6.3 ± 0.1^b^
T2_E	68.4 ± 0.5	14.7 ± 2.3^b^	81.5 ± 0.1^b,c^	1.5 ± 0.0^b,d^
T1_WB	69.2 ± 1.2	11.6 ± 0.1^b^	75.9 ± 0.5	6.5 ± 0.0^b^
T2_WB	69.3 ± 1.2	9.5 ± 0.3^b,c^	77.0 ± 0.4	2.0 ± 0.1^b,d^
T1_MP	68.8 ± 1.0	10.1 ± 0.5^b^	76.2 ± 0.4	6.5 ± 0.0^b^
T2_MP	69.6 ± 0.8^a^	10.3 ± 0.1^b^	77.6 ± 0.6	1.9 ± 0.1^b,d^
T1_S	67.7 ± 0.1	13.3 ± 0.4^b^	79.6 ± 0.7	6.4 ± 0.0^b^
T2_S	67.9 ± 0.1	11.7 ± 0.2^b^	79.8 ± 1.8	1.6 ± 0.0^b,d^

FW, fresh weight; DM, dried matter.

^a^Indicates a significant difference between chicken patties without fat substitution (CTRL) and those with fat substitution (*p* < 0.05).

^b^Indicates a significant difference between chicken patties without fat substitution (CTRL) and those with fat substitution (*p* < 0.005).

^c^Indicates a significant difference between chicken patties with 50% fat substitution (T1) and those with 100% fat substitution (T2) (*p* < 0.05).

^d^Indicates a significant difference between chicken patties with 50% fat substitution (T1) and those with 100% fat substitution (T2) (*p* < 0.005).

Similar results were observed when freeze-dried mushroom powders were added as potential fat replacers (Table 5). Moisture content of the mushroom powder substituted patties (59.9 to 62.4%) was comparable to the control (CTRL_M). Contrarily, mushroom powder substituted patties had significantly lower cooking weight losses of 4.9 to 7.7% than CTRL_M (*p* < 0.05). Moreover, the chicken patties with fat substitution had significantly lower crude fat content (1.5 to 15.9%) than CTRL_M (*p* < 0.005). Likewise, chicken patties at 100% fat substitution with dried mushroom powders (T2_M) had a higher moisture retention, lower cooking loss, and higher protein content than those at 50% fat substitution (T1_M). Protein content of mushroom powder substituted patties were similar to CTRL_M (54.3%), however, T2_M samples have a marginally higher protein content at 55.8 to 63.4%. The highest protein content was from substitution with mini portobello and white button (*A. bisporus* group).

**TABLE 5:** Chemical composition (g/100 g DM), cooking loss (%) and protein content of different chicken patties without fat substitution (CTRL_M), as well as at 50% (T1_M) and 100% (T2_M) fat substitution with freeze-dried mushroom powders.

	Moisture (%)	Cooking loss (%)	Protein (g/100 g DM)	Crude fat (g/100 g DM)
CTRL_M	60.7 ± 3.0	13.7 ± 4.8	54.3 ± 3.5	22.5 ± 3.9
T1_OM	59.9 ± 2.5	7.7 ± 5.3	53.7 ± 3.7	15.3 ± 1.0^b^
T2_OM	61.9 ± 0.9	5.2 ± 2.8^a^	55.8 ± 4.8	1.6 ± 0.1^b^,c
T1_EM	61.2 ± 0.9	5.5 ± 0.9^a^	54.3 ± 0.5	15.1 ± 1.4^b^
T2_EM	62.4 ± 0.3	5.3 ± 1.6^a^	56.1 ± 2.0	1.6 ± 0.3^b,c^
T1_WBM	61.4 ± 0.6	6.0 ± 2.3^a^	54.4 ± 3.7	15.9 ± 0.3^b^
T2_WBM	62.2 ± 0.4	5.3 ± 1.0^a^	59.5 ± 4.5	2.0 ± 0.3^b,c^
T1_MPM	62.3 ± 1.0	5.4 ± 1.4^a^	57.3 ± 2.5	15.7 ± 0.5^b^
T2_MPM	62.4 ± 1.0	4.9 ± 1.4^a^	63.4 ± 1.2	1.5 ± 0.3^b,c^
T1_SM	61.4 ± 1.6	5.7 ± 3.4^a^	53.2 ± 3.0	15.2 ± 1.7^b^
T2_SM	61.5 ± 1.4	5.1 ± 2.4^a^	58.9 ± 1.1	1.9 ± 0.3^b,c^

DM, dried matter.

^a^Indicates a significant difference between chicken patties without fat substitution (CTRL_M) and those with fat substitution (*p* < 0.05).

^b^Indicates a significant difference between chicken patties without fat substitution (CTRL_M) and those with fat substitution (*p* < 0.005).

^c^Indicates a significant difference between chicken patties with 50% fat substitution (T1) and those with 100% fat substitution (T2) (*p* < 0.005).

### 3.3. Physical qualities of mushroom extract and powder patties

The increase in hardness and chewiness of patties appeared to be directly proportionate to the amount of β-glucan extract added but varied based on the type of mushrooms (Table 6). Shrinkage of T1 and T2 extract substituted patties (−7.3% to 14.3%) were lower than CTRL (17.1%). The hardness and chewiness of T1 patties ranged from 24.3 to 61.8 N and 8.6 to 33.1 N, respectively, whereas in T2, hardness and chewiness ranged from 26.7 to 60.5 N and 9.4 to 34.5 N, respectively. Remarkably, among the five mushrooms, the addition of β-glucan polysaccharide extract from oyster mushroom did not increase the hardness of chicken patty despite its extract containing the highest amount of β-glucan content (29.8 g/100 g DM). Instead, the addition of oyster mushroom extracts in the chicken patties displayed a lower hardness and chewiness profile as compared to CTRL at 26.9 N and 13.2 N. In contrast, the addition of β-glucan extract from mini portobello increased the hardness of chicken patty by two times (Table 6).

**TABLE 6:** Texture analysis of the chicken patties without fat substitution (CTRL), as well as at 50% (T1) and 100% (T2) fat substitution with β-glucan polysaccharide extracted from different mushrooms.

	Original dimensions (cm × cm × cm)	Final dimensions (cm × cm × cm)	Shrinkage (%)	Hardness (*N*)	Chewiness (*N*)
CTRL	2.8 × 2.8 × 1.2	2.6 × 2.5 × 1.2	17.1	26.9 ± 6.6	13.2 ± 2.3
T1_O	3.1 × 3.1 × 1.2	2.8 × 2.8 × 1.4	4.8	24.3 ± 2.6	8.6 ± 1.6
T2_O	3.1 × 3.0 × 1.1	2.8 × 2.8 × 1.4	−7.3	26.7 ± 2.8	9.4 ± 0.8
T1_E	2.8 × 2.8 × 1.2	2.7 × 2.8 × 1.3	−4.5	46.6 ± 7.2	19.5 ± 5.5
T2_E	2.8 × 2.7 × 1.3	2.7 × 2.7 × 1.3	3.6	47.7 ± 6.8	19.1 ± 3.7
T1_WB	3.0 × 3.0 × 1.2	2.9 × 2.9 × 1.1	14.3	47.3 ± 6.0	21.2 ± 2.6
T2_WB	3.0 × 3.0 × 1.3	3.0 × 2.9 × 1.2	10.8	50.2 ± 2.7	24.5 ± 0.01
T1_MP	3.0 × 3.0 × 1.3	3.0 × 3.0 × 1.3	0.0	61.8 ± 9.1	33.1 ± 6.5
T2_MP	2.8 × 2.9 × 1.2	2.8 × 2.8 × 1.2	3.4	58.0 ± 11.9	34.5 ± 5.6
T1_S	2.7 × 2.7 × 1.3	2.6 × 2.7 × 1.3	3.7	43.4 ± 4.6	23.2 ± 2.4
T2_S	2.8 × 2.8 × 1.3	2.7 × 2.7 × 1.3	7.0	60.5 ± 0.8^a^	26.6 ± 4.3

^a^Indicates a significant difference between chicken patties with 50% fat substitution (T1) and those with 100% fat substitution (T2) (*p* < 0.05).

Shrinkage of mushroom powder patties were relatively low (−5.2 to 15.9%) as compared to CTRL_M (18.8%). Similar hardness and chewiness results were observed in mushroom powder patties as compared to CTRL_M (85.5 N hardness, 44.7 N chewiness), with the hardness ranging from 68.0 to 145.0 N and chewiness ranging from 27.7 to 74.9 N (Table 7). The addition of mushroom powder modified the color of the chicken patties. Chicken patty at 100% fat substitution with mini portobello powder had the highest lightness (L*) of 25.3, and the lowest redness (a*) of 3.9 and yellowness (b*) of 5.8. The color difference from CTRL_M based on ΔE, where T1_EM (10.0) had the lowest color shift and T2_MPM had the greatest difference (41.4) (Table 7).

**TABLE 7:** Texture analysis and color analysis of the chicken patties without fat substitution (CTRL_M), as well as at 50% (T1_M) and 100% (T2_M) fat substitution with freeze-dried mushroom powders.

	Original dimensions (cm × cm × cm)	Final dimensions (cm × cm × cm)	Shrinkage (%)	Hardness (N)	Chewiness (N)	Color analysis			
						L*	a*	b*	Delta (E)
CTRL_M	7.9 × 8.0 × 0.9	7.5 × 7.7 × 0.8	18.8	85.5 ± 11.7	44.7 ± 6.9	62.5 ± 1.1	3.9 ± 0.7	24.1 ± 0.5	NA
T1_OM	7.5 × 7.5 × 1.0	7.2 × 7.3 × 1.0	6.6	68.0 ± 16.0	27.7 ± 2.5	53.7 ± 2.3	8.5 ± 2.3	26.9 ± 1.9	10.3
T2_OM	7.4 × 7.5 × 1.0	7.3 × 7.4 × 0.9	12.4	92.8 ± 10.8	41.9 ± 12.1	50.4 ± 1.3	6.8 ± 0.9	23.6 ± 0.3	12.4
T1_EM	7.4 × 7.7 × 1.0	7.3 × 7.4 × 1.0	5.2	80.4 ± 6.3	46.3 ± 10.1	53.1 ± 5.9	7.1 ± 2.1	24.4 ± 1.7	10.0
T2_EM	7.4 × 7.4 × 0.9	7.1 × 7.1 × 1.0	−2.3	107.0 ± 15.3	50.9 ± 16.3	49.3 ± 1.9	7.6 ± 0.9	24.5 ± 1.1	13.8
T1_WBM	7.6 × 7.6 × 0.9	7.3 × 7.3 × 1.0	−2.5	112.0 ± 9.4	55.1 ± 6.7	39.2 ± 0.6	6.6 ± 0.6	16.2 ± 1.4	24.8
T2_WBM	7.4 × 7.4 × 1.0	7.1 × 7.2 × 1.0	6.6	117.0 ± 14.7	54.4 ± 7.3	31.6 ± 2.1	5.9 ± 0.2	11.1 ± 1.6	33.5
T1_MPM	7.4 × 7.5 × 0.9	7.3 × 7.4 × 0.9	2.7	125.0 ± 19.7	62.4 ± 9.4	33.8 ± 1.6	5.6 ± 1.0	11.5 ± 2.9	31.4
T2_MPM	7.5 × 7.5 × 1.0	7.1 × 7.1 × 1.0	10.4	145.0 ± 19.5^b^	74.0 ± 9.9^a^	25.3 ± 1.8	3.9 ± 0.9	5.8 ± 1.5	41.4
T1_SM	7.5 × 7.5 × 1.0	7.2 × 7.3 × 0.9	15.9	98.1 ± 29.1	60.5 ± 19.0	48.1 ± 0.9	6.4 ± 0.7	20.9 ± 1.9	15.0
T2_SM	7.4 × 7.5 × 0.9	7.2 × 7.3 × 1.0	−5.2	135.6 ± 23.2^a^	74.9 ± 12.1^a^	43.4 ± 2.6	6.3 ± 0.7	18.0 ± 1.3	20.2

^a^Indicates a significant difference between chicken patties without fat substitution (CTRL_M) and those with fat substitution (*p* < 0.05).

^b^Indicates a significant difference between chicken patties without fat substitution (CTRL_M) and those with fat substitution (*p* < 0.005).

L*, Lightness; a*, Redness; b*, Yellowness.

### 3.4. Fatty acid profile of mushroom extract and powder patties

**FIGURE 2:**
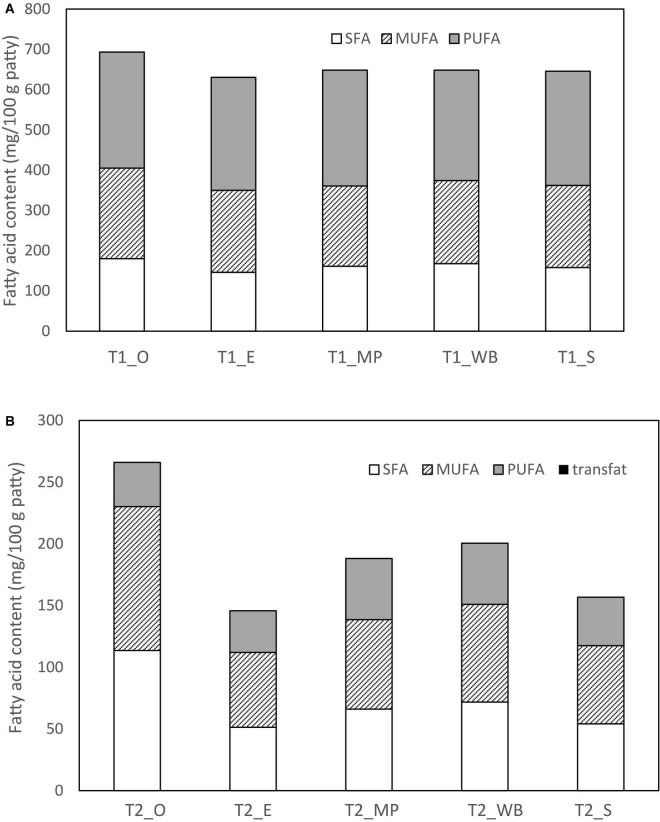
Fatty acid content (mg/100 g patty DW) of the chicken patties at **(A)** 50% (T1) and **(B)** 100% (T2) fat substitution with β-glucan polysaccharide extracted from different mushrooms.

CTRL had the highest levels of C16, C18, C18:1, C18:2, and C18:3 (n-3) fatty acids than T1 (50% fat substitution) and T2 (100% fat substitution) samples (Table 8). For the mushroom extract patties, T1 patties at with oyster mushroom polysaccharide extract (T1_O) had the highest amount of SFA (180.0 mg/100 g patty), MUFA (225.0 mg/100 g patty), and PUFA (288.0 mg/100 g patty) (Table 8 and Figure 2). As compared to CTRL, all extract substituted patties had lower SFA, majority were comparatively higher in MUFA and PUFA. T1 chicken patties substituted with β-glucan polysaccharide extracted from mini portobello (T1_MP) and shiitake mushroom (T1_S) had lower amounts of SFA (161.0 and 158.0 mg/100 g patty, respectively), but higher MUFA (199.0 and 204.0 mg/100 g patty, respectively), and PUFA (288.0 and 284.0 mg/100 g patty, respectively). T2 patties substituted with β-glucan polysaccharide extracts with oyster mushroom (T2_O) had the highest amount of SFA (114.0 mg/100 g patty) and MUFA (117.0 mg/100 g patty), but *A. bisporus* groups T2_MP and T2_WB had the highest amount of PUFA (49.5 and 49.6 mg/100 g patty, respectively) (Table 8 and Figure 2).

**TABLE 8:** Fatty acid profiles (mg/100 g DM patty) of the chicken patties without fat substitution (CTRL), as well as at 50% (T1) and 100% (T2) fat substitution with β-glucan polysaccharide extracted from different mushrooms.

Fatty acid	CTRL	T1_O	T2_O	T1_E	T2_E	T1_MP	T2_MP	T1_WB	T2_WB	T1_S	T2_S
C14	1.9 ± 0.3	2.2 ± 0.7	2.4 ± 0.2^b^	1.2 ± 0.1	1.0 ± 0.0^b^	1.6 ± 0.0^a^	1.3 ± 0.1^b^	2.3 ± 0.1	1.2 ± 0.0^b,c^	1.4 ± 0.2	1.1 ± 0.1^b^
C15	ND	ND	0.4 ± 0.0	ND	0.2 ± 0.0	ND	0.2 ± 0.0	ND	0.3 ± 0.0	ND	0.2 ± 0.0
C16	143.0 ± 6.8	125.0 ± 6.7^a^	80.8 ± 3.1^b^	99.4 ± 3.6	36.2 ± 0.7^b^	109.0 ± 0.7	45.7 ± 0.1^b^	110.0 ± 1.9	49.1 ± 0.5^b,c^	102.0 ± 0.0	38.1 ± 0.9^b,c^
C17	1.4 ± 0.1	ND	0.8 ± 0.5	ND	0.2 ± 0.0	0.9 ± 0.1	0.4 ± 0.0	1.0 ± 0.3	0.4 ± 0.1	7.3 ± 0.1	0.3 ± 0.0
C18	58.8 ± 3.6	50.2 ± 10.0	29.2 ± 1.8^b,d^	42.9 ± 7.4	13.5 ± 0.3^b,d^	46.1 ± 1.2	18.1 ± 0.5^b,d^	51.1 ± 2.9	20.3 ± 0.4^b,d^	44.0 ± 4.8	14.3 ± 0.4^b,d^
C20	4.1 ± 0.5	2.6 ± 1.2	ND	2.7 ± 0.4	0.2 ± 0.0^b,d^	3.1 ± 0.1	0.3 ± 0.1^b,d^	3.2 ± 0.1	0.4 ± 0.1^b,d^	2.9 ± 0.6	0.1 ± 0.0^b,d^
SFA	210.0 ± 11.1	180.0 ± 18.6	114.0 ± 0.7^b,d^	146.0 ± 11.3	51.3 ± 1.1^b,d^	161.0 ± 1.8	66.0 ± 0.6^b,d^	168.0 ± 4.5	71.7 ± 1.1^b,d^	158.0 ± 5.1	54.2 ± 1.4^b,d^
C14:1	ND	ND	0.3 ± 0.0	ND	0.2 ± 0.0	ND	0.2 ± 0.0	ND	0.2 ± 0.0	ND	0.2 ± 0.0
C16:1	6.6 ± 0.9	8.5 ± 1.6	5.3 ± 5.2	6.3 ± 0.0	3.3 ± 3.4^a,c^	7.4 ± 0.5	6.1 ± 0.3	6.9 ± 0.3	6.1 ± 0.0	6.8 ± 0.6	3.9 ± 3.9^a,c^
C18:1	269.0 ± 0.7	216.0 ± 4.2	110.0 ± 4.0^b,d^	197.0 ± 2.2	56.4 ± 1.0^b,d^	190.0 ± 0.3	65.2 ± 1.2^b,d^	196.0 ± 0.6	71.8 ± 1.1^b,d^	195.0 ± 5.4	58.4 ± 1.4^b,d^
C20:1	2.8 ± 0.1	ND	1.5 ± 0.7	ND	0.8 ± 0.0^b^	2.2 ± 0.6	1.0 ± 0.0	3.2 ± 0.5	1.1 ± 0.0^a,d^	2.4 ± 1.0	0.9 ± 0.1^b,c^
MUFA	278.4 ± 0.1	225.0 ± 2.6	117.0 ± 8.5^b,d^	203.0 ± 2.2	60.7 ± 2.4^b,d^	199.0 ± 0.8	72.6 ± 0.8^b,d^	206.0 ± 0.4	79.2 ± 1.1^b,d^	204.0 ± 5.7	63.3 ± 2.4^b,d^
C18:2	44.1 ± 0.8	255.0 ± 18.4^b^	33.8 ± 8.2^d^	254.0 ± 14.6^b^	28.1 ± 0.5^d^	249.0 ± 4.6^b^	41.0 ± 0.1^d^	238.0 ± 4.9^b^	40.8 ± 1.3^d^	251.0 ± 8.6^b^	33.0 ± 0.8^d^
C18:3n6	0.4 ± 0.0	ND	ND	ND	0.2 ± 0.0	ND	0.3 ± 0.1	ND	0.3 ± 0.0	ND	0.5 ± 0.0
C18:3n3	3.4 ± 0.3	33.3 ± 2.5^b^	2.1 ± 1.0^d^	26.1 ± 1.1^b^	2.6 ± 0.0^d^	31.2 ± 1.7^b^	3.4 ± 0.0^d^	27.1 ± 0.8^b^	3.2 ± 0.3^d^	30.0 ± 2.0^b^	2.7 ± 0.1^d^
C20:2	1.1 ± 0.3	ND	ND	ND	0.7 ± 0.3	1.6 ± 0.2	1.2 ± 0.1	1.8 ± 0.1	1.6 ± 0.0	ND	0.6 ± 0.1
C20:3n6	0.9 ± 0.1	ND	ND	ND	0.7 ± 0.0	1.9 ± 0.0	1.3 ± 0.0	2.1 ± 0.1	1.2 ± 0.1	ND	0.9 ± 0.3
C20:4	2.4 ± 0.0	ND	ND	ND	1.5 ± 0.6	4.5 ± 0.1	2.3 ± 0.2	4.9 ± 0.1	2.5 ± 0.3	3.0 ± 0.1	1.7 ± 0.0
PUFA	52.3 ± 0.7	288.0 ± 15.9^b^	35.9 ± 9.1^d^	280.0 ± 13.5^b^	33.7 ± 1.3^d^	288.0 ± 2.6^b^	49.5 ± 0.2^d^	274.0 ± 4.2^b^	49.6 ± 1.9^d^	284.0 ± 10.7^b^	39.3 ± 1.0^d^
C18:1t	ND	ND	ND	ND	0.3 ± 0.0	ND	0.3 ± 0.1	ND	0.5 ± 0.1	ND	0.3 ± 0.1

^a^Indicates a significant difference between chicken patties without fat substitution (CTRL) and those with fat substitution (*p* < 0.05).

^b^Indicates a significant difference between chicken patties without fat substitution (CTRL) and those with fat substitution (*p* < 0.005).

^c^Indicates a significant difference between chicken patties with 50% fat substitution (T1) and those with 100% fat substitution (T2) (*p* < 0.05).

^d^Indicates a significant difference between chicken patties with 50% fat substitution (T1) and those with 100% fat substitution (T2) (*p* < 0.005).

**FIGURE 3:**
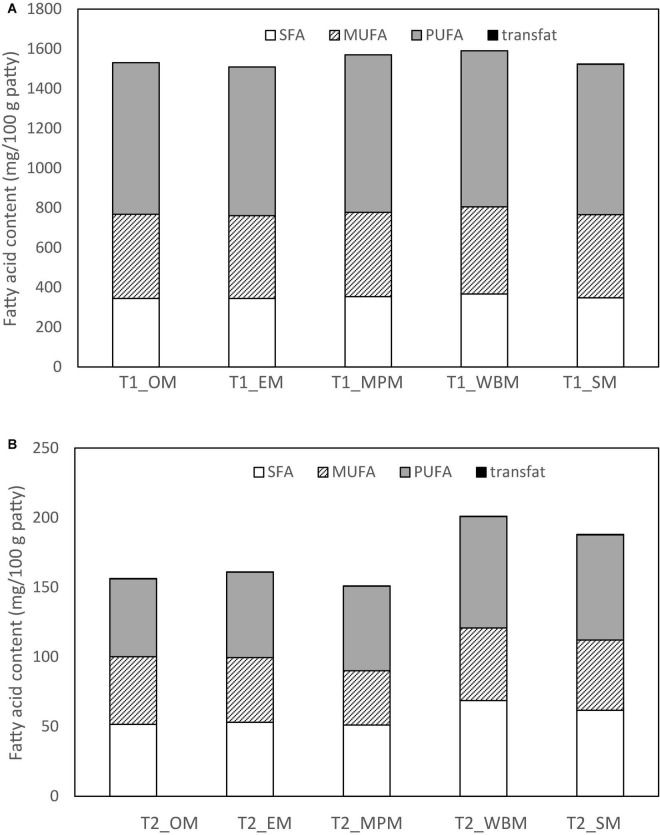
Fatty acid content (mg/100 g patty) of the chicken patties at **(A)** 50% (T1_M) and **(B)** 100% (T2_M) fat substitution with freeze-dried mushroom powders.

Evidently, all fat substituted patties with mushroom powder (T1_M and T2_M) were lower in SFA than the control (CTRL_M). However, all patties had a lower MUFA and PUFA profile than CTRL_M. For T1_M patties (50% fat substitution), patties substituted with white button mushroom powder (T1_WBM) had the highest amount of SFA (367.0 mg/100 g patty) and MUFA (438.0 mg/100 g patty), while patties substituted with mini portobello (T1_MPM) had the highest amount of PUFA (793.0 mg/100 g patty) (Table 9 and Figure 3). For T2_M patties with full fat substitution with mushroom powder, T2_WBM had the highest amount of SFA (68.6 mg/100 g patty), MUFA (438.0 mg/100 g patty), and PUFA (79.9 mg/100 g patty). A markedly finding was the presence of marginal amounts of trans fats in 100% fat substitution with mushroom powder patties (T2_M) ranging from 0.2 to 1.8 mg/100 g (Table 9 and Figure 3).

**TABLE 9:** Fatty acid profiles (mg/100 g DM patty) of the chicken patties without fat substitution (CTRL_M), as well as at 50% (T1_M) and 100% (T2_M) fat substitution with freeze-dried mushroom powders.

Fatty acid	CTRL_M	T1_OM	T2_OM	T1_EM	T2_EM	T1_MPM	T2_MPM	T1_WBM	T2_WBM	T1_SM	T2_SM
C12	0.7 ± 0.1	0.5 ± 0.1	0.1 ± 0.0	0.6 ± 0.1	0.3 ± 0.0	0.5 ± 0.1	0.1 ± 0.0	0.5 ± 0.1	0.1 ± 0.0	0.5 ± 0.1	0.1 ± 0.0
C14	2.9 ± 0.5	2.0 ± 0.2	0.6 ± 0.1^b,d^	2.4 ± 0.3	1.1 ± 0.1^a,d^	2.2 ± 0.3	0.6 ± 0.0^b,d^	2.2 ± 0.3	0.9 ± 0.1^b,d^	2.1 ± 0.3	0.7 ± 0.1^b,d^
C15	0.5 ± 0.1	0.5 ± 0.1	0.4 ± 0.1	0.3 ± 0.2	0.3 ± 0.0	0.5 ± 0.2	0.4 ± 0.0	0.6 ± 0.2	0.6 ± 0.0	0.5 ± 0.2	0.5 ± 0.1
C16	334.0 ± 7.9	224.0 ± 2.6	34.0 ± 1.1^b,d^	223.0 ± 7.6	34.8 ± 0.9^b,d^	227.0 ± 7.5	31.8 ± 0.4^b,d^	237.0 ± 12.1	42.7 ± 1.0^b,d^	226.0 ± 4.1	41.2 ± 3.1^b,d^
C17	2.5 ± 0.7	1.5 ± 0.2	0.2 ± 0.1^b,c^	1.5 ± 0.3	0.2 ± 0.0^b^	1.7 ± 0.3	0.3 ± 0.0^b,d^	1.8 ± 0.3	0.5 ± 0.0^b,d^	1.5 ± 0.3	0.3 ± 0.0^b,d^
C18	151.0 ± 13.5	97.9 ± 5.1	15.9 ± 0.6^b,d^	98.1 ± 7.2	15.9 ± 0.2^b,d^	101.0 ± 8.2	15.6 ± 0.5^b,d^	105.0 ± 10.2	21.1 ± 0.7^b,d^	99.2 ± 6.7	18.5 ± 0.6^b,d^
C20	12.6 ± 1.4	7.5 ± 0.8	0.2 ± 0.0^b,d^	7.4 ± 0.8	0.1 ± 0.0^b,d^	8.3 ± 1.1	0.9 ± 0.2^b,d^	8.6 ± 1.0	1.2 ± 0.1^b,d^	7.6 ± 0.9	0.2 ± 0.0^b,d^
C21	ND	ND	ND	ND	ND	ND	0.1 ± 0.0	ND	0.2 ± 0.0	ND	ND
C22	13.3 ± 2.0	7.7 ± 0.9	ND	8.0 ± 0.9	ND	8.8 ± 1.3	0.9 ± 0.4	8.8 ± 1.0	0.9 ± 0.0	7.6 ± 0.9	ND
C24	3.8 ± 0.7	2.5 ± 0.5	0.3 ± 0.1^b,d^	2.4 ± 0.5	0.5 ± 0.1^b,d^	2.7 ± 0.6	0.4 ± 0.1^b,d^	2.5 ± 0.6	0.5 ± 0.1^b,d^	2.4 ± 0.6	0.3 ± 0.1^b,d^
SFA	521.0 ± 26.5	344.0 ± 9.3	51.6 ± 1.9^b,d^	344.0 ± 15.7	53.1 ± 0.9^b,d^	353.0 ± 18.5	51.1 ± 0.3^b,d^	367.0 ± 25.4	68.6 ± 0.3^b,d^	347.0 ± 13.9	61.7 ± 3.3^b,d^
C14:1	ND	ND	0.1 ± 0.0	ND	0.1 ± 0.0	ND	ND	ND	0.1 ± 0.0	ND	ND
C16:1	10.1 ± 1.8	8.0 ± 0.8	4.2 ± 0.7^b,d^	8.6 ± 1.1	4.8 ± 0.6^b,d^	7.2 ± 1.4	3.5 ± 0.4^b,d^	8.8 ± 1.4	5.1 ± 0.6^b,d^	8.2 ± 0.6	4.5 ± 1.2^b,d^
C18:1	599.0 ± 7.9	411.0 ± 5.7	43.7 ± 1.6^b,d^	403.0 ± 10.3	40.9 ± 1.6^b,d^	412.0 ± 3.6	34.9 ± 0.8^b,d^	425.0 ± 10.7	46.3 ± 3.7^b,d^	405.0 ± 6.9	45.2 ± 2.2^b,d^
C20:1	7.4 ± 1.8	5.2 ± 0.5	0.6 ± 0.1^b,d^	5.1 ± 0.5	0.6 ± 0.1^b,d^	5.5 ± 0.6	0.5 ± 0.1^b,d^	4.6 ± 2.1	0.7 ± 0.1^b,d^	5.3 ± 0.5	0.7 ± 0.0^b,d^
MUFA	616.0 ± 6.3	424.0 ± 6.0	48.6 ± 2.1^b,d^	417.0 ± 10.3	46.4 ± 2.1^b,d^	424.0 ± 1.9	38.9 ± 0.8^b,d^	438.0 ± 9.9	52.2 ± 4.3^b,d^	418.0 ± 6.4	50.4 ± 3.5^b,d^
C18:2	944.0 ± 41.8	655.0 ± 15.2	45.2 ± 1.5^b,d^	635.0 ± 23.5	41.3 ± 1.5^b,d^	679.0 ± 28.4	48.9 ± 0.9^b,d^	686.0 ± 27.4	65.2 ± 3.5^b,d^	647.0 ± 20.9	61.3 ± 5.1^b,d^
C18:3n6	ND	ND	0.2 ± 0.0	ND	0.2 ± 0.1	ND	0.2 ± 0.0	ND	0.3 ± 0.0	ND	0.5 ± 0.1
C18:3n3	147.0 ± 11.7	91.3 ± 8.3	1.6 ± 0.3^b,d^	96.9 ± 10.1	9.7 ± 0.4^b,d^	95.5 ± 9.9	1.5 ± 0.1^b,d^	80.0 ± 36.7	1.8 ± 0.3^b,d^	92.1 ± 9.0	2.4 ± 0.7^b,d^
C20:2	2.7 ± 0.9	2.3 ± 0.5	0.9 ± 0.2	2.1 ± 0.5	1.0 ± 0.1	2.4 ± 0.3	1.0 ± 0.2	2.4 ± 0.5	1.4 ± 0.2	2.3 ± 0.3	1.3 ± 0.3
C20:3n6	3.4 ± 0.9	2.8 ± 0.5	1.6 ± 2.0	2.9 ± 0.3	1.9 ± 0.1	3.1 ± 0.3	1.6 ± 0.1	3.3 ± 0.2	2.3 ± 0.1	3.2 ± 0.6	1.8 ± 0.2
C20:4	13.7 ± 5.2	11.3 ± 2.6	5.9 ± 3.1^b,c^	11.3 ± 2.6	7.2 ± 0.9^b,c^	12.7 ± 1.3	7.1 ± 0.3^b,c^	12.6 ± 2.2	8.9 ± 1.1^b,c^	12.0 ± 2.4	7.6 ± 1.0^b,c^
C20:5	ND	ND	ND	ND	ND	ND	ND	ND	ND	ND	ND
C22:6	ND	ND	0.4 ± 0.1	ND	ND	ND	0.5 ± 0.1	ND	ND	ND	0.5 ± 0.1
PUFA	1111.0 ± 24.5	763.0 ± 5.4	55.9 ± 2.9^b,d^	749.0 ± 18.4	61.3 ± 2.4^b,d^	793.0 ± 16.8	60.8 ± 0.6^b,d^	785.0 ± 33.4	79.9 ± 4.5^b,d^	757.0 ± 9.9	75.5 ± 6.7^b,d^
C18:1t	ND	ND	0.2 ± 0.1	ND	0.2 ± 0.0	ND	0.2 ± 0.0	ND	0.3 ± 0.0	1.8 ± 0.8	0.4 ± 0.1

^a^Indicates a significant difference between chicken patties without fat substitution (CTRL_M) and those with fat substitution (*p* < 0.05).

^b^Indicates a significant difference between chicken patties without fat substitution (CTRL_M) and those with fat substitution (*p* < 0.005).

^c^Indicates a significant difference between chicken patties with 50% fat substitution (T1) and those with 100% fat substitution (T2) (*p* < 0.05).

^d^Indicates a significant difference between chicken patties with 50% fat substitution (T1) and those with 100% fat substitution (T2) (*p* < 0.005).

## 4. Discussion

### 4.1. Mushroom composition and β-glucan content

Mushrooms are high in moisture and do provide a reasonable protein content. However, deviations in the proximate composition of mushrooms vary by their cultivar, country of origin and the production processes (16). Mushroom polysaccharide of different species vary in its functionality strengths, for example, shiitake mushroom contain β-glucan polysaccharide Lentinan that have a stronger antioxidant activity and amplified effect on reducing cancer cells than other species (7). Indubitably, broad variances in β-glucan content was observed across the five mushrooms investigated in this study. Mushrooms possess both water insoluble and soluble β-glucan in varying levels depending on the mushroom species group (33). The β-glucan content in the mushroom polysaccharide extracts obtained were within previously reported for oyster mushroom (15.3 to 41.5 g/100 g DM), enoki (18.3 g/100 g DM), shiitake (19.8 to 30.4 g/100 g DM), and *A. bisporus* group (6.59 to 13.2 g/100 g DM) (18, 33, 34). Yet, the extracted β-glucan polysaccharide yield was lower than results from previous studies which also utilized UAE on *A. bisporus* (5.91%) and *Tremella mesenterica* (8.26%) but comparable to *Ganoderma lucidum* extracts obtained from hot water extraction method at 1.52% (10, 22). This was probably due to the structural differences of the β-glucan branching, affecting the solubility of β-glucans in each mushroom species during extraction. A previous study comparing shiitake extracts by soxhlet extraction with commercial extracts found that 10% of soxhlet extract contained β-glucan, however, commercial sources contained higher amounts of β-glucan and results varied based on the β-glucan assay used (35). Factors that affect the yield during extraction include UAE power, time of extraction, and extraction temperature (36).

Interestingly, the extracted polysaccharide from mushrooms had a lower β-glucan content than whole mushroom counterparts. This was evident for oyster mushroom and shiitake mushroom where the β-glucan content was only half and a quarter of what was present in its freeze-dried form. The reduction of β-glucan content is probably attributed to the removal of water-insoluble β-glucan during extraction as only the supernatant from the UAE extraction was retained (16). Moreover, there was no additional pre-treatment steps to remove other impurities present in the crude polysaccharide which could include α-glucans, proteins, minerals, and indigestible starches (37, 38). Nevertheless, although no pre-treatment of mushrooms such as soaking in alkali sodium hydroxide or washing with ethanol was performed, our extract had a higher β-glucan content than pre-treated *Ganoderma lucidum* mushroom extract which ranged from 4 to 8 g/100 g fresh weight (39). It should be noted that despite the higher β-glucan content in freeze-dried mushroom than in crude polysaccharide extract, polysaccharide extracts had shown enhanced bioactivity of β-glucan properties along with antioxidants, anti-tumor effects and a reduction in obesity biomarkers when fed to mice (16, 40, 41). The unique structure of mushroom β-glucan linkages also promote anti-cancer properties and its high molecular weight compared to other β-glucan sources increases its bioactivity (42).

### 4.2. Substitution of fat with β-glucan vs. mushroom powder in chicken patties

Remarkably, the addition of increased amounts of β-glucan polysaccharide extract in the patties exhibited higher protein content, but lower cooking losses and lower crude fat content. Results were consistent to previous findings of polysaccharide fortification in fortified cheese where it was observed to have a higher moisture retention, lower protein, and fat content than control cheese (43). Although texture analysis showed that hardness and chewiness of β-glucan substituted patties were generally enhanced with increasing amounts of β-glucan added, palatability of these β-glucan substituted patties may not necessarily be negatively affected. Previously, the addition of β-glucan from oat into beef burgers as a fat replacer have shown favorable sensory results based on its color, aroma, texture, and juiciness, although liking was dependent on the amount of β-glucan added. Nevertheless, the addition of β-glucan reduced the overall cholesterol in the cooked beef burgers and can aid in the development of a functional food with improved nutritional profile (44). Previously, shiitake β-glucan polysaccharides have been added into rice noodles, which improved its dough matrix, noodle structure, texture and cooking retention (7). Moreover, β-glucan from other sources have been successfully used as a food additive such as prebiotics, thickeners, gelling agents, and stabilizers for maintaining food structure in a wide range of food products including dairy products, meat products and bread. Overall, our results support that mushroom β-glucan can provide a high-water holding capacity that can enable food products to increase its moisture retention and reduce cooking loss.

Alike β-glucan substituted patties, mushroom powder patties exhibited similar changes in weight, crude fat, and protein content as compared to its control. However, mushroom powder patties fared better in moisture retention than β-glucan polysaccharide patties. Additionally, color analysis of the mushroom powder patties were favorable. The results for lightness in chicken patties with oyster mushroom powder obtained in this study were similar to results from a previous study where 25 and 50% of chicken was substituted with ground oyster mushroom (26). Moreover, a further study found that there were no changes in acceptability amongst the 60 untrained sensory panel from the added oyster mushroom in chicken patties (45). Yahya and Ting (46) also found significant color acceptance amongst five chicken patties samples with 15 to 60% substitution with fresh oyster mushroom as compared to its control. In addition, 45% substituted patties had the highest sensory score for its overall acceptability across all samples including the control. Generally, mushroom substituted meat patties are well accepted by consumers (13).

Dimensional differences are proportionate to temperature conditions, which are affected by placement of the patties on the pan, and sarcomere shrinkage. Lower fat meat patties have a higher shrinkage in length and height (47). Comparing β-glucan substituted patties to mushroom powder patties, the latter had a more desirable structure retention and texture results, which may be advantageous in consumer acceptance. Previous sensory analyses on low fat meat products expressed mixed critiques, dependent on the type and amounts of substitute used in place of fat. Nevertheless, drivers of consumer meat consumption are the healthier products, socio-economic status, and convenience (48). Therefore, the inclusion of β-glucan or mushroom powder in reducing the fat content of meat patties can increase health properties, while the short cooking time of the patties facilitates consumer convenience. Further work can include sensory tests to determine consumer acceptance and liking.

Chemical composition of patties is affected by processing. Taking protein content into account, the highest from whole mushroom powder were obtained from *A. bisporus* groups. However, when the mushroom polysaccharide extract was added into the patties, the resulting protein content was not proportional, with enoki mushroom attaining the highest protein content. Reasons for this could be attributed to ethanol denaturation or solubility variability due to temperature fluctuations, despite the extraction was conducted on freeze-dried samples that improves protein retention (49).

Broadly, our study showed that the addition of mushroom β-glucan extract and mushroom powders into chicken patties allowed for moisture and structure retention whilst decreasing overall crude fat levels and maintaining protein content. Moreover, oral consumption of mushroom β-glucan polysaccharides in diabetic rats of previous studies have shown to be effective in reducing triglycerides, improve insulin resistance, decrease cholesterol, and lower blood glucose levels implying that the use of β-glucan polysaccharide can be beneficial for decreasing diabetic markers (50). Our results were also in agreement to previous studies which supported the addition of dried oyster mushroom powder in meat patties was favorable to reduce cooking loss, maintained moisture retention and texture profile, while lowering fat content (51).

### 4.3. Fatty acid profile

Mushrooms contain all types of fatty acids with a larger proportion of PUFAs and MUFAs (3, 52). It was expected that the CTRL samples would have the highest fatty acid profile since soybean oil was used as the fat source in the chicken patty formulation in this study. As we reported previously, the major fatty acids in soybean oil included C16 (13.2%), C18 (5.65%), C18:1 (25.2%), C18:2 (46.3%), and C18:3n3 (7.87%) (28).

Interestingly, *A. bisporus* powder substituted patties had the highest fatty acid contents in terms of MUFA and PUFA levels. The variations in the fatty acid profile of the chicken patties substituted with 50% (T1_M) or 100% (T2_M) of freeze-dried mushroom powder were likely due to the differences in mushroom species. Quantities of fatty acid in mushrooms differed by its cultivation processes (53), and the region (3). Mushrooms cultivated in Asia have a higher C18:3n6 content than those cultivated in America, and the highest quantification of MUFA as compared to mushrooms cultivated in other continents. A particular finding was that a small amount of fatty acid C18:1t (elaidic acid) was present in 100% substituted (T2 samples) polysaccharide extract patties with enoki, mini portobello, white button and shiitake mushrooms. The presence of trans fatty acids implied a formation of a double bond in the trans configuration and can be naturally found in small amounts in animal meats or may be attributed from cooking at high temperatures (54).

Overall, the results suggest that the addition of β-glucan crude extracts or mushroom powders increase the PUFA content and the availability of essential fatty acids C18:3n6 and C18:3n3 that can only be obtained from diets and are beneficial in reducing cardiovascular diseases and cancer prevention (55). Both formulations of chicken patties can be further validated through *in vitro* and *in vivo* analyses to validate the nutritional qualities and fatty acid profiles after digestion and its beneficial properties in humans.

## 5. Conclusion

In summary, the five types of commonly consumed mushrooms have a high moisture content ranging from 86.4 to 93.6%, but they have low β-glucan polysaccharide yield of 1.2 to 2.0%. β-glucan content from the crude polysaccharide extract obtained by UAE and ethanol precipitation ranged from 3.8 to 15.9 g/100 g with the highest β-glucan content obtained from oyster mushroom. The inclusion of β-glucan polysaccharide and dried mushroom powder in chicken patties improved the moisture retention, maintained cooking yield, and preserved the overall structure of the food matrix. The increment of hardness and chewiness of the fat substituted chicken patties with β-glucan extracts and dried mushroom powder were varied by the type of mushroom and the amount added into the patties. Crude fat content was lower in both mushroom polysaccharide and extract patties than its control. Moreover, dried mushroom powder may be a better fat replacer than mushroom β-glucan extract in meat products due to increased reduction of crude fat content. White button and mini portobello are two promising mushrooms that can be added into meat patties in powder form due to its excellent MUFA and PUFA content. Further investigations are needed to validate the efficacy of mushroom β-glucan polysaccharide as a fat replacer for meat patties for commercialization. Future applications of utilizing mushroom powder as a method to replace fat in other food products may be a useful strategy in our pursuit of developing low fat functional foods.

## Data availability statement

The original contributions presented in this study are included in this article/Supplementary material, further inquiries can be directed to the corresponding author.

## Author contributions

CH and XB: conceptualization. CS, HL, and MY: methodology and investigation. CS and XB: validation, formal analysis, and writing—original draft preparation. CS, HL, MY, XB, and CH: writing—review and editing. CH: supervision and project administration. All authors contributed to the article and approved the submitted version.
