# Supplementary material for: Is mushroom polysaccharide extract a better fat replacer than dried mushroom powder for food applications?
Source: Front Nutr. 2023 Feb 3;10:1111955. doi: 10.3389/fnut.2023.1111955 (PMC9935597; doi:10.3389/fnut.2023.1111955)
Supplement: Supplementary file 1 [file Data_Sheet_1.DOCX]

**Supplementary Materials**

Is mushroom polysaccharide extract a better fat replacer than dried mushroom powder for food applications?

Cheryl Jie Yi See Toh^1^, Xinyan Bi^1^, Hui Wen Lee^1^, Michelle Ting Yun Yeo^1^, Christiani Jeyakumar Henry^1,2*^

^1^Clinical Nutrition Research Centre (CNRC), Singapore Institute of Food and Biotechnology Innovation (SIFBI), Agency for Science, Technology and Research (A∗STAR), Singapore, Singapore

^2^Department of Biochemistry, Yong Loo Lin School of Medicine, National University of Singapore, Singapore, Singapore

*** Correspondence:**Professor Christiani Jeyakumar Henry
jeya_henry@sifbi.a-star.edu.sg

**Calculations of hardness (N) and chewiness (N)**

Hardness refers to the maximum force of the first compression in the double-bite test. Chewiness is calculated as hardness × cohesiveness × springiness. Cohesiveness is the area under the curve of the second compression divided by the area under the curve of the first compression. Springiness is the time taken during the second compression divided by the time taken in the first compression (S1).


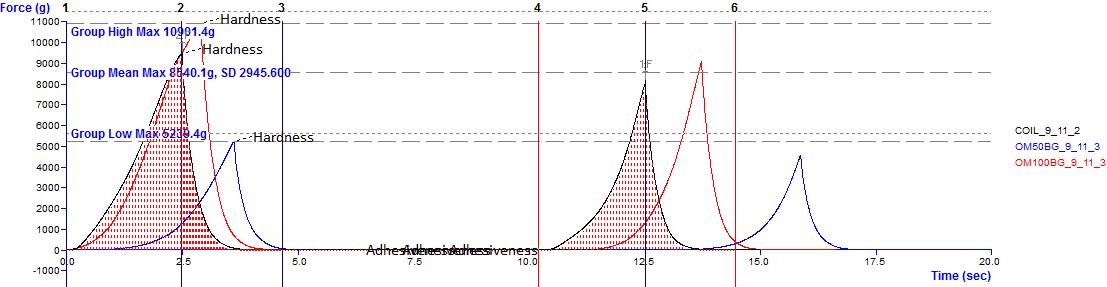


Fig S1. Two bite texture analysis test graph for CTRL (in black) against T1_OM (in blue) and T2_OM (in red).


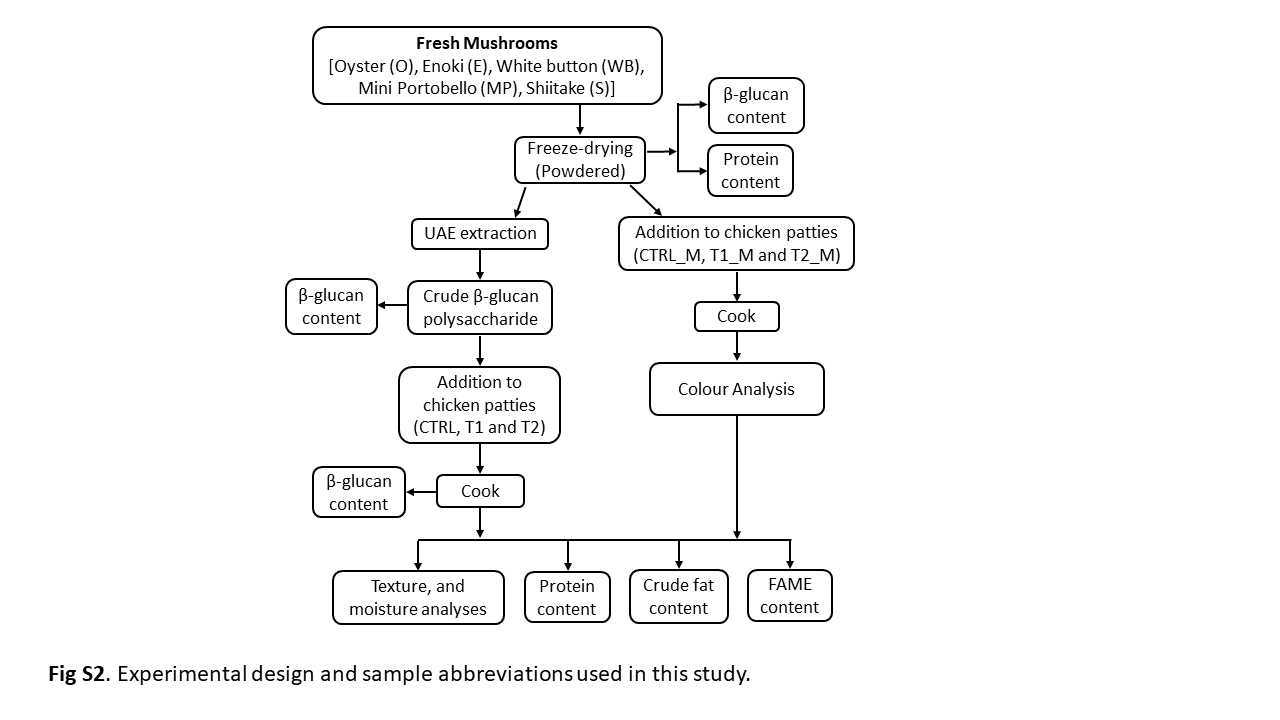


**References**

S1. Texture Technologies Corp (2022). Overview of TPA Hamilton, MA: Texture Technologies Corp.; [Retrieved from: <https://texturetechnologies.com/resources/texture-profile-analysis#tpa-measurements>.
